# The health implications of distrust in the food system: findings from the dimensions of trust in food systems scale (DOTIFS scale)

**DOI:** 10.1186/s12889-021-11349-9

**Published:** 2021-07-28

**Authors:** Emma Tonkin, Trevor Webb, Julie Henderson, Paul R. Ward, John Coveney, Samantha B. Meyer, Dean McCullum, Annabelle M. Wilson

**Affiliations:** 1 College of Medicine and Public Health, Flinders University, Bedford Park, South Australia 5042 Australia; 2 Behavioural & Regulatory Analysis Section, Food Standards Australia New Zealand, Majura Park, Australian Capital Territory 2609 Australia; 3 College of Nursing and Health Sciences, Flinders University, Bedford Park, South Australia 5042 Australia; 4 School of Public Health and Health Systems, University of Waterloo, 200 University Ave West, Waterloo, Ontario N2L 3G1 Canada; 5 Food and Controlled Drugs Branch, Public Health Services, Public Health and Clinical Systems, SA Health, 11 Hindmarsh Square, Adelaide, South Australia 5000 Australia

**Keywords:** Food, Food industry, Health behaviour, Survey, Trust

## Abstract

**Background:**

Consumer trust in food systems is essential for consumers, food industry, policy makers and regulators. Yet no comprehensive tool for measuring consumer trust in food systems exists. Similarly, the impact that trust in the food system has on health-related food behaviours is yet to be empirically examined. The aim of this research was to develop a comprehensive instrument to measure trust in the food system (the Dimensions of Trust in Food Systems Scale (DOTIFS scale) and use it to explore whether trust in the food system impacts consumers’ health-related behaviours.

**Methods:**

The DOTIFS scale was developed using sociological theories of trust and pre-existing instruments measuring aspects of trust. It was pilot tested and content validity was assessed with 85 participants. A mixed-methods exploration of the health-related behaviours of 18 conveniently sampled Australian consumers with differing trust scores determined by the DOTIFS scale was then conducted. During March–July 2019 shopping- and home-observations were used to assess participants’ food safety practices and exposure to public health fortification programs, while the CSIRO Healthy Diet Score determined their adherence to national dietary guidelines.

**Results:**

The DOTIFS scale was found to have high comprehension, ease of use and content validity. Statistical analysis showed scale scores significantly trended as predicted by participants’ stated level of trust. Differences were found in the way individuals with more or less trust in the food system comply with national dietary guidelines, are exposed to public health fortification programs, and adhere to recommended food safety practices.

**Conclusions:**

The DOTIFS scale is a comprehensive, sociologically- and empirically- informed assessment of consumer trust in food systems that can be self-administered online to large populations and used to measure changes in consumer trust over time. The differences in health-related behaviours between individuals with varying levels of trust warrant further investigation.

**Supplementary Information:**

The online version contains supplementary material available at 10.1186/s12889-021-11349-9.

## 1. Background

Due to the critical role played by diet in health and disease, governments and world authorities globally invest in the development of dietary guidelines [1–3], mandatory fortification of foods [4], and setting and enforcing food safety standards in food production and retail environments [5]. Additionally, public healthy eating campaigns continue to be funded worldwide, from large-scale national media campaigns, to small local community programs [6]. In Australia, the current Health Star Rating system and the previous national social marketing campaign ‘Go for 2&5’ are examples of the substantial investment made by the Australian Government in supporting consumers to make choices for optimal health. However, the information from the scientific community is challenged by alternative and pseudo-health professionals as well as non-health trained health bloggers, celebrities and conspiracy theorists through the rise in Web 2.0 [7]. These groups cast doubt on the legitimacy of government messages and activities, particularly through questioning areas of scientific uncertainty and progress, broadly contrasting ‘new ways’ (perceived scientific intrusion in food production; pesticides, genetic modification, food additives) as dangerous and ‘old ways’ (unpasteurised foods, organic processes) and ‘natural’ foods as safe. In combination with increased distance between food producers and consumers [8], recent food safety incidents, and public awareness of unethical conduct in food production through incidents like the Horsemeat scandal [9, 10], this strategy is effective in undermining consumer trust in food systems [7, 11]. The Food system in this paper is taken to mean the methods by which food ingredients move from production; processing and distribution; and consumption [12]. The various steps in the food system give rise to different forms of trust and distrust by consumers. For example, in Australia [12], the UK [13] and across Europe [14] consumers report that pesticides, additives, artificial sweeteners, genetic modification and other forms of biotechnology in food continue to be high priority personal food safety concerns. Thus, for consumers what constitutes safe, healthy and ethically appropriate food, and whether to trust in conventional food systems to provide it, continues to be privately and publicly contested.

It is theorised that distrust in food systems may have consequences for consumer decision-making, particularly food choice and acceptance of expert advice [15]. The avoidance of foods perceived to be unsafe or risky (for example, gluten or red meat) may lead to the exclusion of entire food groups from the diet, without medical advice to do so [16]. If done without appropriate dietary substitutions, often requiring fortified products which are commonly themselves distrusted, this can compromise the nutritional adequacy of the diet. In Australia, organic food products are exempt from some mandatory fortification, therefore consumer avoidance of conventional food products and the exclusive use of organic products may also reduce exposure to the national fortification of bread flours with folate and thiamine, salt with iodine and tap water with fluoride. These public health fortification programs have all been shown to significantly reduce deficiencies and associated disease incidence at the population level [17–19]. Indeed, distrust in the chlorination and fluoridation of tap water has been suggested to be linked with the movement towards drinking highly filtered or bottled water, contributing to rising childhood dental caries [20]. These types of food behaviours are particularly concerning for nutritionally vulnerable groups such as children and pregnant women; commonly those who public health campaigns seek to protect. Additionally, Ekici [21] found that distrusting consumers are more likely to shop outside of conventional food systems, relying instead on farmers markets and local produce. While this in and of itself is not problematic, and indeed may result in increased dietary variety and motivation to use fresh produce, there can be unintended consequences of the practices found within some alternative markets. For example, engagement with alternative markets and their associated community groups may increase access to products like unpasteurized milk, consumption of which is linked to outbreaks of food borne illness, and has resulted in deaths [22, 23]. Additionally, previous research has also shown there to be a group of consumers, typically with fewer financial and social resources, who despite verbally expressing distrust in food do not have the resources to access farmers’ markets and other alternative food markets. They therefore control their family’s food intake in other, also potentially risky, ways such as ‘going without’ other staple food items to afford the higher price of organic produce in supermarkets [24]. Yet while the theoretical case for trust impacting food choices and subsequently health outcomes is convincing, to date no research has empirically and comprehensively examined the link between consumers’ trust in food systems and their food choices, food safety practices, and health outcomes.

Partially this gap in knowledge is due to the complexity around measuring consumer trust in food. Instruments currently exist that include some components for measuring trust in food systems. The Edelman Trust barometer is an annual global report on various states and conditions of trust, typically in governments and business, but occasionally with a focus on food and beverages [25]. Similar large scale surveys include the European Commission’s Eurobarometer [14], the UK Food Standards Agency’s Food and You Survey [26] and tracker [13], the Food Standards Australia and New Zealand Consumer Attitudes surveys [27, 28], the US Food and Health survey [29], and the Australian survey of Social Attitudes [30]. All these instruments however balance multiple data collection agendas and subsequently they do not comprehensively assess all dimensions of trust in food. They generally focus on a single dimension, like trust in regulatory bodies or industry, and so include only a few questions related to trust in food. Additionally, these surveys are typically not informed by sociological theories of trust.

A handful of academic studies have been conducted using instruments that aim to more comprehensively measure trust in food [15, 31, 32], notably Poppe and Kjaernes [31] exploration of trust in food in Europe. However, while this survey was sociologically underpinned it was not designed to be self-completed, reducing its efficiency when deployed in very large populations and its ability to be deployed quickly in response to changes in the food environment (e.g., a food incident). It also may have limited relevance to a global population due to the specification of food products incorporated within it. More recently, Benson et al. developed a toolkit to measure six aspects of trust across the food chain. These are: trust in organisations, product trust, interpersonal trust, trust in the food chain, organisation distrust, and general distrust [33]. The scales from this tool can be used independently or as a whole. The Organisation for Economic Co-operation and Development (OECD) recently released guidelines for the measurement of trust generally, but this did not extend to a focus on trust in food [34]. As such, while existing instruments provide a useful starting point, there is currently no comprehensive, sociologically informed, self-completed, quickly deployed instrument for the measurement of consumer trust in food and food systems.

Knowledge about the impact of trust in food systems on health would be powerful in two ways. First, public health campaigns with a food choice or safety focus typically aim to address lack of knowledge, positioning consumer education as central to success. However, if distrust in food systems is driving undesirable food choices and food safety practices, this ‘knowledge fix’ approach [35–37] will need to be positioned alongside strategies for building food system trust to counter risky food behaviours. Second, an Australian study [38] which presented issues on consumer food trust in relation to food governance actors and regulators found that due to the lack of research linking trust in food systems and concrete health outcomes, these actors consider distrust in the food system to be a ‘personal psychological issue’ and therefore not of public concern, or importantly, action. This position underrates the potential implications of trust in food systems, or lack thereof, for the success of existing substantial investments in public health. Therefore, if it is shown that there are risky health behaviours associated with distrust in food systems, programs to better address food trust become important, and what have up to now been considered ‘personal psychological issues’ become public health imperatives.

The aims of this paper are;

1. to develop a comprehensive, sociologically informed, self-completed instrument to measure consumer trust in the food system, and
2. to explore whether trust in the food system impacts
  - adherence to national dietary guidelines,
  - exposure to public health fortification programs, and
  - adherence to recommended food safety practices.

## 2. Methods

### 2.1. Study design

**Fig. 1:**
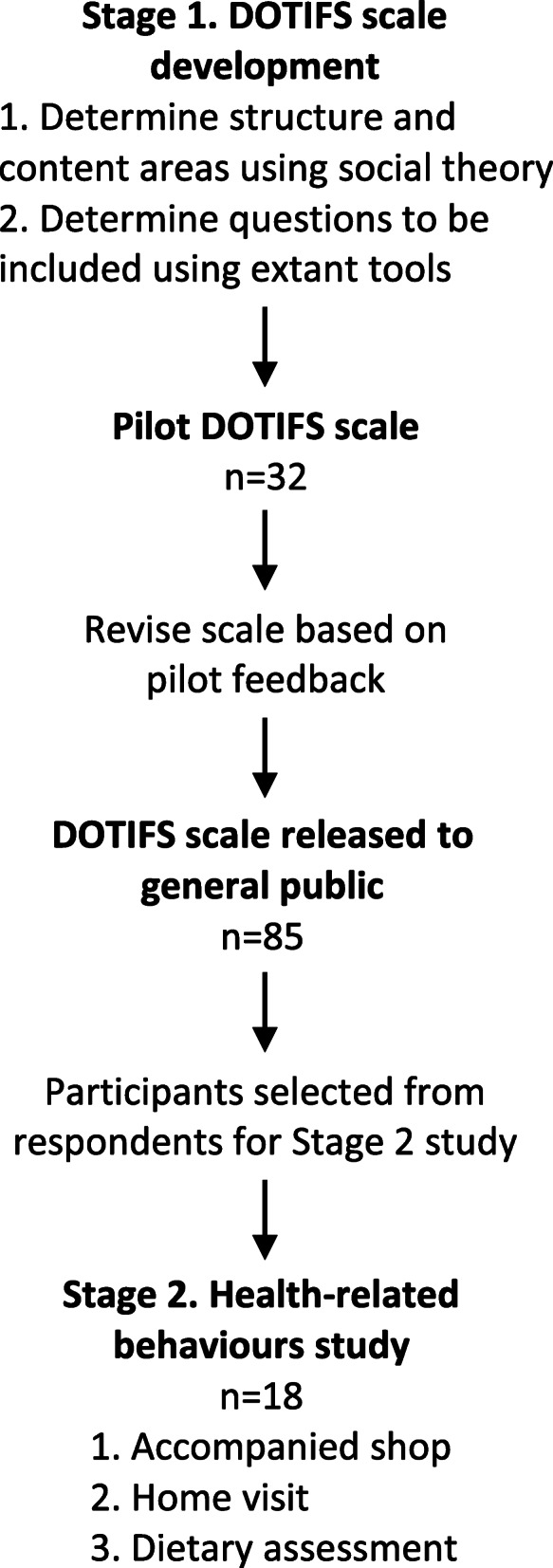
Study methodology

The research involved two stages (Fig. 1):

1. The development of an online instrument to measure trust in the food system (hereafter the ‘Dimensions of Trust in Food Systems Scale (DOTIFS scale)’, and
2. A mixed-methods exploration of the food practices of consumers with differing levels of trust in the food system, to explore their adherence to national dietary guidelines and recommended food safety practices, and their exposure to public health fortification programs (hereafter “health-related behaviours’).

Ethics approval for all components of the research reported in this manuscript was granted by the Flinders University Social and Behavioural Research Ethics Committee (project number 8229).

#### 2.1.1. Stage 1. DOTIFS scale development

The development of the DOTIFS scale was carried out in 2 stages: determining the structure and content areas to be covered within the instrument (see ‘Development of DOTIFS scale structure and content areas’ section); selecting questions to be used to measure content areas (see ‘Questions chosen for inclusion’ section) (see Fig. 1). To determine the structure and content areas, the team’s expertise with regards to sociological trust theory was utilised. Previous research demonstrated that Australian consumers were inclined to trust the Australian food regulatory system unless they had had experienced failure in food safety (eg: habitual trust) but were distrusting of media messaging about food due to competing claims leading to self-reliance in food decision-making [39–41]. The specific questions used within content areas were then chosen through the review of existing instruments that quantitatively measure either trust generally, trust in food systems, trust in food actors, or food attitudes and beliefs that can relate to the formation of trust judgements [42] such as beliefs and attitudes around food safety. Given the Australian setting of this research, instruments developed for the Australian population were particularly considered, as were those specifically developed for global generalisability. While a large number of instruments, questionnaires and surveys were reviewed, questions considered potentially relevant were ultimately extracted from 12 sources [13–15, 25, 26, 28–32, 34, 43].

##### 2.1.1.1. Development of DOTIFS scale structure and content areas

A sociological definition of trust was used to structure the design of the DOTIFS scale to ensure it comprehensively measured all dimensions of consumer trust in the food system. Trust is,

‘a particular level of subjective probability with which an agent *[consumers]* assesses that another agent *[food industry, regulatory/governance actors]* or group of agents *[the food system]* will perform a particular action *[fulfil the expectations consumers hold]* and in a context which affects his *[sic]* own action *[food decision making].*’ [44] (p. 217)

Therefore, the overarching themes to be considered were: A) food system agents/actors, B) consumer expectations and, C) consumer food decision-making. These themes are similar to those proposed by Poppe and Kjaernes ( [31], p. 31) in their development of their instrument to measure trust in food; ‘*trust – in whom – with regards to what*’. That is, when thinking about measuring trust in food it is important to structure empirical efforts around determining not only the level of trust (‘*trust’*), but also the key concepts of whom the trust is placed in (‘*in whom’*) and about what specifically consumers are trusting (‘*with regards to what’*). Each overarching theme was then considered in the context of extant food-trust literature to determine what elements would need to be included for a comprehensive assessment of each. The themes are now described below:

A) Food system actors is typically conceptualised straightforwardly, and therefore translated to a content area titled *Trust in Food System Organisations and Institutions*. However, given there is a sociological distinction between identifiable actors and the systems they are part of [45], a second domain titled *Belief in the System* was also included.

B) Consumer expectations have been measured directly through questions relating to the safety and integrity of the food supply, as well as indirectly by measuring the level of concern or worry consumers express about specific food issues. Therefore, content areas titled *Food Safety and Integrity* and *Food Concerns* were included.

C) Consumer food decision-making in relation to food trust has previously been measured indirectly by determining what actions consumers take that are thought to reflect distrust, therefore the domain *Engagement with Food Issues* was included. Finally, following the OECD recommendation that all measurements of trust should include some measurement of generalised trust [34], the domain *Generalised Trust* was included. To ensure a logical structure and flow to the instrument, and taking into consideration the need to place more emotive/biasing questions towards the end to ensure they do not influence less-emotively based questions, the content areas were structured into the DOTIFS scale domains as follows:

- Domain 1. Generalised Trust.
- Domain 2. Food Safety and Integrity.
- Domain 3. Trust in Food System Organisations and Institutions.
- Domain 4. Food Concerns.
- Domain 5. Engagement with Food Issues and Activism.
- Domain 6. Belief in the Food System.

##### 2.1.1.2. Questions chosen for inclusion

The existing trust and food attitudes instruments used as question sources [13–15, 25, 27–32, 34, 43] were then reviewed, and all potentially relevant questions extracted into a single document organised by the domain headings of the DOTIFS scale. The questions within each domain were then interrogated to assess duplication of concepts measured and the theoretical completeness of the domain overall. Where questions measured the same concept/idea (duplicates), questions previously used or validated in an Australian population were selected. Where neither were previously used in an Australian population, the question thought to have the most face-validity was selected. Where all questions in the domain were used but the domain was thought to be theoretically incomplete, a new question was developed. See Table 1 for a summary of the source and rationale for the questions included in each domain.

**Table 1:** Summary of the source and justification of each domain of the DOTIFS scale

Domain	Source of questions	Rationale for inclusion
*Generalised trust*	From the *OECD Guidelines on Measuring Trust* [34] with adaptions based on use in an Australian population in [43].	The *OECD Guidelines on Measuring Trust* [34] recommends always including a measure of generalised trust when assessing trust
*Food Safety and Integrity*	Adapted from the UK Food Standards Agency 2018 tracker [13, 26] and Edelman Trust Barometer [25].	Trust – in whom – with regards to ***what*** (please see ‘Development of DOTIFS scale structure and content areas’ section) Perceptions of food safety and extent to which food governance reflects consumer values/issues beyond safety.
*Trust in Food System Organisations and Institutions*	Overarching question structures adapted from Poppe and Kjaernes, Trust in food in Europe, from the Edelman trust barometer ‘Overall Trust’ measure for companies and *OECD Guidelines on Measuring Trust* [25, 31, 34].	Trust – ***in whom*** – with regards to what Measures consumer trust in food institutions and the organisation that make up the food system.
*Food Concerns*	Overarching question structures adapted from the Food Fears survey [15], Poppe and Kjaernes, Trust in food in Europe [25] and the UK Food Standards Agency 2018 tracker [13, 26]. Individual items within grouped questions from Eurobarometer [14], FSANZ [28], the Food and Health Survey [29], and Taylor et al. [32].	Trust – in whom – with regards to ***what*** Measures degree of concern with food issues.
*Engagement with Food Issues and Activism*	Adapted from Poppe and Kjarnes [25] and the UK Food Standards Agency 2018 tracker [13, 26]	***Trust*** – in whom – with regards to what Measures extent of actions in relation to food concerns, which reflects (dis) trust
*Belief in the Food System*	Adapted from the ‘System is Failing’ measure from Edelman Trust Barometer [25]	***Trust*** – in whom – with regards to what Measures social justice, hope for food system, confidence and desire for change
*Demographics*	Adapted from [30, 32, 43].	Demographics shown to be relevant to trust and used in the context of trust assessment in the cited sources.

##### 2.1.1.3. Scoring system

Domains 2–6 were included in the scoring to determine an overall ‘trust in the food system’ score. Given all questions within the tool were measured on a 1–7 scale (excluding the Engagement and Activism domain), scores for each domain were summed and divided by the number of questions within the domain to arrive at an overall domain score of 1–7; one reflecting complete distrust and seven complete trust (for example, each participant receives a Food Concerns domain score of between 1 and 7). This ensured equal weighting of all questions within a domain. For the Engagement and Activism domain, ‘yes’ responses were scored one point, while ‘no’ responses were scored seven points, and the domain score was calculated as previously described. To again ensure equal weighting of each content area, the five domain scores were summed to calculate the overall trust score, and as the lowest possible score for each domain was 1, five points were subtracted from the total to give an overall possible score range of 0–30; zero reflecting complete distrust and thirty complete trust.

##### 2.1.1.4. Pilot and content validity testing of the DOTIFS scale

The DOTIFS scale was then formatted into an online survey using Qualtrics, an online survey delivery tool (Utah, USA), the first page incorporating a question ensuring informed consent, and piloted through March and April 2019 with 32 experts and members of the researchers’ networks to determine comprehension and ease of use, as well as whether they felt the instrument captured trust status adequately (content/construct validity, hereafter referred to as content validity). Modifications were made in an iterative manner throughout the pilot in response to feedback. While comprehension and ease of use questions were excluded from the final DOTIFS scale tested with the public, content validity questions remained. The final DOTIFS scale instrument used in the following stages of this research is provided in Additional file 1.

Content validity was then further tested through deployment of the instrument with the general public. Recruitment for this testing was conducted in South Australia from April–July 2019, and sought to sample a diverse range of participants > 18 years of age. Recruitment utilised flyers in supermarkets, libraries, farmers’ markets, gym change rooms and on community notice boards, a method previously used with success [11, 46]. Additionally, Facebook posts in initially public (e.g. University Alumni) and increasingly targeted private groups (e.g. thrifty shopping mothers) were used to advertise the DOTIFS scale survey link. Recruitment materials asked the public to independently complete the survey online via a provided link, with the final page of the survey outlining the Stage 2 mixed-methods study and requesting people leave their contact details if interested in participating further.

#### 2.1.2. Stage 2 health-related behaviours study

Mixed-methods were then used to explore the relationship between trust score on the DOTIF scale and adherence to government recommendations in relation to nutrition and food safety. The employment of both qualitative and quantitative research provides a better understanding and insight into the relationship between trust and food choice and intake.

##### 2.1.2.1. Sampling and recruitment

Participants who had completed Stage 1 and had left their details were contacted to organise a time to complete the Stage 2 study and placed into categories of trust status based on their overall trust score. For ease of reporting, and acknowledging the recommendations regarding labelling individuals as trusting or distrusting [34], the more trusting group will henceforth be referred to as ‘trusters’, the ‘on the fence’ group ‘uncertain’ and the less trusting group ‘distrusters’. Participants with an overall trust score > 16.85 were placed in the ‘trusters’ group, 13.15–16.85 were placed in the ‘uncertain’ and < 13.15 placed in the ‘distrusters’ group, utilising a 12.5% range around the midpoint for determining the cut-points. Given these cut-points, although informed by the early pilot data, were arbitrary, consideration was also given to participants’ own reported level of trust (Additional file 1, ValidityQuestion6) when grouping, particularly for scores close to cut-points. All participants who left their details were followed up for observation until no new themes emerged regarding food choice reasoning and justification for health-related behaviours within each participant group (data saturation) [47], which resulted in 18 participants, six within each group.

##### 2.1.2.2. Data collection

Data collection involved three components: an accompanied shop [48], a home observation and a dietary assessment. A researcher accompanied participants on a regular food shop at their predominant shopping location to determine the ‘where, why and how’ of their food choice considerations. During the shop the researcher interviewed participants regarding their food choice considerations and shopping practices, while also assessing the overall shopping environment. Following this, the researcher accompanied participants to their home for an observation of the visible food safety practices within their food preparation areas, as well as an interview to discuss their usual food handling, storage, and cooking practices. Finally, the researcher assisted participants to complete the CSIRO Healthy Diet Score survey [49]. The CSIRO Healthy Diet Score survey is an extension of the Short Food Survey [50] from which was developed a Dietary Guideline Index score reflecting a reported diet’s compliance with the Australian Dietary Guidelines. The survey has been described in detail elsewhere [49], but includes 38 questions covering the core food groups as well as fluid and discretionary food intakes. Scoring compares reported intake with age and gender-specific recommended intakes, with each component score out of 10 (except discretionary intake which is score out of 20), and the total diet score calculated by summing individual component scores (maximum possible score 100). This survey was chosen as it and the scoring systems have been validated in Australian adults [51]. Hand-written notes during the accompanied shop and home visit were used to record environmental observations. All conversation during the accompanied shop, home visit and dietary assessment were audio recorded, with total data collection time ranging from 2.5–4 h per participant. Written informed consent was provided by all participants, and participants were reimbursed $25 each for costs associated with participating.

##### 2.1.2.3. Analysis

A data entry form designed around the study aims of assessing food choice considerations (e.g. shopping location), exposure to food fortification programs (e.g. use of tap or filtered water) and food safety practices (e.g. defrosting practices) was developed in Qualtrics (Utah, USA). Audio recordings and hand-written notes were used to enter data by the researcher after each observation. These data, each participant’s DOTIFS scale data, and their dietary data were imported into and linked in IBM SPSS Statistics version 25 (IBM Corp, Armonk, NY, USA) and organised by trust status group. Numerical data were then quantitatively described and analysed using ANOVA and non-parametric equivalents where appropriate. Text data were qualitatively analysed using thematic analysis, with specific focus on drawing out negative cases to provide depth and nuance of understanding [52] and theoretical insights into, if there exist differences in health-related behaviours between the groups, and how these are related to trust in the food system.

## 3. Results

### 3.1. Stage 1. DOTIFS scale development

We begin by first reporting the comprehension, ease of use and completion data from both the pilot and general public deployment of the DOTIFS scale, followed by reporting content validity findings.

#### 3.1.1. Comprehension, ease of use, completion

Pilot feedback indicated that comprehension and ease of use of the DOTIFS scale were overall high, with some exceptions. Early feedback suggested the terms ‘food industry’, ‘consumer values’ and ‘food safety’ needed to be substituted and/or explicitly defined. Pilot participants also recommended some minor changes to the wording of questions 9, 19 and 21. Several participants reported having trouble responding to the Belief in the System domain as it was a different perspective on how they typically thought about food issues, but not to the point that it should be excluded from the tool as they felt it measured a unique aspect of their trust not otherwise captured. Finally, the response options used for questions in the pilot DOTIFS scale were those used in the original instruments/questionnaires from which they came, and therefore there was diversity in the length of scale used between questions (e.g. 5 discreet responses vs 0–10 scale). Feedback suggested it was important that the same scale be used consistently and formatting all response options to a 1–7 scale or yes/no where possible would achieve the best balance of data sensitivity and respondent fatigue.

#### 3.1.2. Sample description for stage 1

Time to fully complete the DOTIFS scale by the general public ranged from 6.68 to 30.85 min, with mean 14.60 ± 6.16 min. Overall 85% of people who consented to begin completed all sections. All (100%) completed the Generalised Trust section and the first of the six food trust sections (Food Safety and Integrity domain), 76 (89%) completed the first two, 73 (86%) completed the first three, and all the remaining 72 (85%) participants completed the full DOTIFS scale. Due to the convenience sampling approach used, few participants had not received formal higher education (*n* = 14, 20%), with 41% (*n* = 30) having a bachelor’s degree and 39% (*n* = 28) reporting to have a degree higher than a bachelor’s. However, Fisher’s exact tests showed no differences in participants’ reports of accuracy of trust and domain scores based on formal education status (all *p* > 0.05).

#### 3.1.3. Content validity

Overwhelmingly participants reported both their overall trust scores and domain scores to be an accurate reflection of their trust. For those who thought their score was not accurate, on only a single occasion this was reported as ‘not at all accurate’ rather than ‘not very accurate’, and this was for the Food Concerns domain. These scores were included in the analysis. The following percentages exclude participants who did not provide an answer about score accuracy.

##### 3.1.3.1. Overall trust scores

Sixty-six (94%) participants thought their Overall Trust Score was accurate, with *n* = 4 (6%) suggesting their score was lower than they had expected. Further follow up of two of these respondents suggested that possibly the tool was accurately measuring their trust (which was low), but in their response to whether they trust the food system the participant was conflating trust and dependence [53], one saying, ‘I mean, I don’t feel good about it but I have no choice but to trust’. Overall trust scores for the 72 participants with complete data were normally distributed, with mean 14.51 (SD 3.17) on a scale of 0–30. Despite a tight overall range of scores (min = 8.54, max = 21.61) a Kruskal-Wallis Test showed the overall trust score trended significantly downwards as predicted by participants’ own reported level of trust (Table 2, *p* < 0.001).

**Table 2:** Overall trust score as determined by the DOTIFS scale, grouped by expressed trust selection

Expressed trust	***n***	Median score (/30)	Min score	Max score
I strongly trust	1	19.06*	19.06	19.06
I mostly trust	35	15.93*	10.90	21.61
I’m undecided, on the fence	15	14.63*	12.24	19.71
I mostly do not trust	21	11.58*	8.54	15.51
I strongly do not trust	0	–	–	–

*Significance of trend *p* < 0.001

##### 3.1.3.2. Individual domain scoring accuracy

Eighty-one (98%) participants thought their Food Safety and Integrity domain score was accurate, while *n* = 2 (2%) reported the score too low. All participants who responded considered their Trust in Food System Organisations and Institutions domain score to be accurate (*n* = 74, 100%). The Food Concerns domain score was the most disputed, with *n* = 61 (87%) participants considering this an accurate reflection of their level of concern. This section was the only to be reverse scored, and it is possible there was some confusion when participants were rating the accuracy of their scores as for the questions the ‘extremely concerned’ score was a 7, while for the overall domain score high concern was represented by 1. Three (4%) participants objected to their scores because of what they saw as diversity in the areas asked about (e.g. Artificial sweeteners vs environmental sustainability); they felt they were mostly unconcerned about the issues presented but felt very strongly about a small number of issues, and were therefore not happy with a mid-range averaged score. Two (3%) other participants simply stated the score was too low, and a third suggested it was too high. Scores for both the Engagement with Food Issues domain *n* = 65 (96%), and Belief in the System domain *n* = 65 (97%) were thought by the large majority to be accurate, with *n* = 3 (4%) and *n* = 1 (3%) participants respectively suggesting their score was too low, the score identifying them as more engaged or sceptical than they think of themselves.

### 3.2. Stage 2 health-related behaviours study

#### 3.2.1. Sample characteristics

Participant characteristics are presented in Table 3. Statistical testing showed the sampling strategy to have been successful, with an ANOVA (F = 58.66, df = 2,14, *p* < 0.001) and post-hoc testing demonstrating the participants selected as trusters had significantly higher mean overall trust scores than those selected as uncertain, who themselves had significantly higher trust scores than those selected as distrusters (Table 3). Two participants were scored as distrusters while reporting ‘I mostly trust’, however these were the participants referred to in ‘Overall trust scores’ section as theoretically conflating trust and dependence.

**Table 3:** Stage 2 Health-related behaviours study participant characteristics

	Gender	Age	Highest level of education	Political affiliation	Predominant shopping location	Expressed trust ^***a***^	Trust score (/30)	Trust score mean (SD)
**Trusters**								
T01	F	25–34	Bachelor’s	None	Supermarket	MT	18.03	17.92 (0.81)*
T02	F	35–44	Higher degree	None	Supermarket	U	18.52	
T03	M	25–34	Higher degree	Greens	Supermarket	MT	17.81	
T04	M	25–34	Higher degree	None	Supermarket	MT	16.83	
T05	M	65+	Secondary	None	Supermarket	ST	19.06	
T06	M	55–64	Higher degree	None	Supermarket	MT	17.28	
**Uncertain participants**								
A01	M	25–34	Diploma/Voc	Labour	Supermarket	U	14.93	14.81 (1.09)*
A02	F	25–34	Bachelor’s	Greens	Markets	U	13.35	
A03	M	65+	Higher degree	None	Multiple	MT	15.38	
A04	F	25–34	Bachelor’s	None	Supermarket	U	14.63	
A06	M	45–54	Year 11	Liberal	Supermarket	MT	16.51	
A07	F	35–44	Bachelor’s	None	Supermarket	U	14.05	
**Distrusters**								
D01	F	55–64	Year 11	None	Multiple	MDNT	10.72	11.81 (1.00)*
D02	F	25–34	Bachelor’s	Greens	Multiple	MDNT	12.19	
D03	M	45–54	Diploma/Voc	None	Supermarket	MT	12.77	
D04	F	35–44	Higher degree	None	Markets	MT	12.97	
D05	M	65+	Higher degree	Liberal	Markets	MDNT	10.61	
D06	F	45–54	Higher degree	Greens	Supermarket	MDNT	11.58	

* all between group differences *p* < 0.001

^a^*Abbreviations*: MT ‘I mostly trust’, *U* ‘I am undecided, on the fence’, *ST* ‘I strongly trust’, *MDNT* ‘I mostly do not trust’

##### 3.2.1.1. Predominant shopping location

There were clear differences in predominant shopping location between the groups (Table 3). Considerations around convenience (in terms of both distance from home, cost and range of products) dominated trusters’ reasoning for predominantly shopping at supermarkets. A number mentioned shopping at independently owned supermarkets to support the local economy, but none said they would rule out shopping at a major supermarket chain.

Four of the uncertain participants shared this reasoning, three with the caveat that they would prefer to shop at independently owned supermarkets if they could afford the cost, while two reported they would never shop at a supermarket because they do not believe their production practices should be supported, from human, animal or environmental welfare perspectives (e.g. fairness in trade with smaller producers, food miles, food waste, excessive use of plastics). These two participants procured their food from local farmers’ markets and/or specialty stores, citing having a relationship or at least direct communication with sellers/producers as important to them.

This was also important to all but two of the distrusters. Three distrusters reported to actively shop exclusively in alternative markets because they believe the food to be safer with respect to production practices (pesticides) and additives (preservatives, emulsifiers). These participants spent considerable time and energy procuring food in this way. A lack of these resources, as well as perceived cost, was reported by the other two distrusters as their reason for ‘having to’ shop at supermarkets, despite the fact that ‘I hate it’ (D03).

##### 3.2.1.2. Food choices

The reasons for between-product food choices (e.g. choice of two types of apple) given by participants were diverse. For trusters, taste and cost predominated, with three mentioning plastic waste and choices to support the local economy as additional considerations. None reported these considerations to be so strong that they would not buy a food they intended to if they could not find a product meeting their criteria, they would simply buy something else that was there (e.g. non-Australian made). This was not the case for three uncertain consumers and five distrusters however, who reported they would either seek out the same product elsewhere (which was why some regularly shopped in multiple locations), or would ‘go without’ if they could not find or afford the product meeting their criteria, which many reported had happened in recent memory. Their food choice criteria were also typically more extensive, with preferences for local produce due to perceived safety, reducing food and plastics waste, and fair trade and free-range. For all participants food labelling use reflected their food choice priorities, many across all groups seeking country-of-origin labelling and the Nutrition Information Panel.

Five trusters reported growing home produce, all for the simple enjoyment of gardening and fostering a connection to food production. Three used conventional pest and soil management practices, with T06 summing up their approach as ‘the more toxic the chemical the better’. All the three uncertain consumers and six distrusters who grew home produce, as well as one of the trusters, reported using organic/low-chemical/no pest and soil management, such as natural remedies and hand weeding. This reflected their reasoning for growing home produce, which was typically to reduce their exposure to strong agricultural chemicals.

#### 3.2.2. Adherence to national dietary guidelines

Other than one participant who had briefly tried vegetarianism, none of the trusters had ever excluded any food group from their diet for non-medical reasons. Two uncertain participants had explored low gluten and excluding dairy, and two further were currently, and one further had previously been, vegetarian or actively reducing meat intake. Two distrusters were actively excluding some dairy foods without medical advice, and one excluding gluten, while a third had previously been vegetarian but had to stop for medical reasons. Universally the reason for choosing a vegetarian/low meat lifestyle was for environmental sustainability reasons, in addition to not believing the medical discourse around eating and not trusting production practices supported animal welfare to the degree they would be happy with. The reasoning for excluding all other food groups were for the perceived health benefits.

Dietary component and overall diet scores are summarised in Table 4, and broadly reflect the qualitative reports given above. A Kruskal-Wallis Test indicated an overall inverse trend of dietary variety with food system trust (Table 4), while a Fisher’s Exact test also revealed that distrusters were more likely to have lower healthy dietary fats scores compared with trusters, although this was borderline significant (*p* = 0.067).

**Table 4:** Dietary assessment

	Individual Food Group Component scores, median (IQR), score /100^a^								
	Vegetable	Fruit	Grains	Meat	Dairy	Discretionary	Fluid	Dietary Variety^**#**^	Overall diet score
**Trusters**	92.14 (69.94, 100.00)	50.00 (1.88, 56.25)	86.31 (50.77, 97.32)	82.06 (49.24, 100.00)	58.90 (22.86, 71.79)	9.62 (0.00, 34.45)	99.76 (98.20, 100.00)	57.67 (55.33, 77.58)^	55.89 (51.50, 70.59)
**Uncertain participants**	95.00 (50.54, 100.00)	75.00 (44.64, 100.00)	62.18 (45.89, 79.61)	48.75 (41.19, 93.43)	40.71 (27.30, 96.00)	25.67 (20.47, 39.27)	100.00 (99.88, 100.00)	70.50 (67.42, 72.17)	61.84 (56.25, 64.54)
**Distrusters**	85.00 (45.77, 100.00)	82.14 (50.00, 100.00)	67.71 (57.20, 85.27)	82.76 (59.64, 100.00)	48.57 (40.53, 58.97)	28.62 (0.00, 49.05)	97.31 (81.82, 100.00)	81.67 (75.17, 82.83)^	63.46 (48.79, 72.98)

^#^ overall trend *p* = 0.02

^^^ median difference 24.00, *p* = 0.01

^a^ scores were not normally distrusted

#### 3.2.3. Exposure to public health fortification programs

All the trusters ate bread that was fortified with folate and iodised salt. One uncertain participant actively chose organic, unfortified breads with ‘proper’ flour (A03), while one distruster chose to make their own bread exclusively. All the trusters drank plain tap water and stated they either did not object to or actively supported fluoridation of tap water. Uncertain participants were evenly split between using tap and filtered water (all had a Puratap which does not filter out fluoride, although the participants were unsure if this was the case). Four distrusters used filtered water; three had chosen to have a filter attached to their kitchen sink, while the fourth used charcoal filters in glass bottles to purify tap water. These four participants commented that they had considered and were unsure about the safety and necessity of chlorinating and fluoridating tap water, although they too were unsure if their filtration system did filter fluoride out (most said they would prefer that it did). A fifth distruster boiled her water, and the sixth went against this trend drinking plain tap water, even commenting that when he had previously lived on a property that ran exclusively on rainwater he had supplemented the household’s water with fluoride. He was however a trained dentist. Only two participants objected to iodine fortification of salt, most commenting that they used so little salt it did not matter to them. One uncertain participant and one distruster actively chose non-iodised salt, citing they had heard of iodised salt being contaminated with microplastics and therefore did not consider it safe.

#### 3.2.4. Adherence to recommended food safety practices

No participants reported ever having actively sought non-pasteurised dairy due to perceived concerns about the health implications of pasteurisation (although some avoided dairy altogether), however one uncertain and two distrusters reported to consume non-pasteurised dairy when they infrequently had access to it. Four participants cultured their own sourdough or yoghurt; one truster and one distruster because they simply enjoyed it and had a science background therefore felt confident in managing the food safety aspects, while one uncertain participant and one distruster due to a scepticism of the safety/quality of equivalent purchased products, but they too were confident of their safety practices. Due to their shopping locations, three of the distrusters were exposed to undesirable food sale practices such as the sale and promotion of bitter apricot kernels and bone broth powders.

While most participants who shopped at supermarkets simply followed the layout of the shop in terms of selecting items - typically vegetables first, then cold foods, meats and the delicatessen, followed by the frozen foods section and finally the dry store aisles – two trusters deliberately shopped non-heat vulnerable products first, with foods requiring refrigeration last, even structuring their shopping lists in this pattern. The other exception was two distrusters who had cold/frozen foods out of cold storage for considerable lengths of time because they shopped at markets and multiple stores, typically far from home. A similar pattern was seen in determining if food was spoiled. All the trusters reported they are primarily guided by date-stamps (both best-before and use-by date stamps), although two said they were not completely governed by them. Only two uncertain consumers reported using date-stamps, two others saying they look but frequently still consume expired products, and two others saying they would never use date-stamps as a guide. Similarly, three distrusters reported being guided but not governed by date-stamps, while three others were vehement that they would not discard food based on date-stamp expiry dates. There were no clear differences in the way any of the groups managed leftovers or defrosting, chopping boards (although more trusters used plastic boards rather than wooden), or fridge stacking. All trusters had soap for handwashing at their kitchen sink, and this was not always the case for uncertain and distrusting consumers.

## 4. Discussion

The DOTIFS scale developed in this research is the first comprehensive and sociologically informed assessment of consumer trust in food systems that can be self-administered online in large populations. Even with content validity questions included, the response duration averaged under 15 min, and the instrument was considered by respondents to be acceptably easy to use and comprehend. Most importantly, an overwhelming majority of participants felt the individual domain scores and overall trust score was a reasonably accurate reflection of their trust in food systems. Statistical analysis also showed scores significantly trended as predicted by participants stated level of trust, therefore we can be confident that population level trends in score reflect true differences in consumer trust in food systems, and the DOTIFS scale can be used as an accurate measure of changes in consumer trust over time.

Similarly, the Stage 2 study suggests there are differences in the way individuals with more or less trust in the food system comply with national dietary guidelines, are exposed to public health fortification programs, and adhere to recommended food safety practices. In brief, distrust of the food system was associated with a greater propensity to source food through markets or independent producers (supporting previous suggestions of this in the literature [11, 21]), purchase food with reference to ethical, animal welfare and environmental concerns and to avoid use of chemicals in food production. Distrust of the food system was also associated with avoidance of foods without medical advice (again supporting theorisations in extant literature [15, 16]); the consumption of products which have not been fortified and lapses in food safety such as consumption of unpasteurised dairy products and extended food purchasing time with frozen goods. Distrusters were also less likely to adhere to best by or use by dates. The benefit of the mixed-methods approach used here is that participants were able to articulate that it was frequently reasoning relating to trust considerations that caused them to make these alternative, and at times risky, choices. However, these qualitative data may also be used to form hypotheses and specific survey questions that could be used in conjunction with the DOTIFS scale to quantitively determine whether these trends persist in a population representative sample.

The trained critical ability of the highly educated sample of this study was advantageous at this early phase of instrument development as it enabled thorough critique of the DOTIFS scale and its content. However, while statistical analysis indicated no differences between formally educated groups in reported accuracy of scores, it means we have fewer perspectives from less formally educated groups around comprehension and ease of use. Nonetheless, the DOTIFS scale has been shown in this study to have considerable potential, and with further investment to conduct larger studies to determine repeatability and acceptability in a sample more representative of the general population, as well as to conduct factor analysis to comprehensively explore its dimensionality, it could have substantial impact in both the regulatory and policy settings.

### 4.1. Research, regulatory and policy futures for the DOTIFS scale

If baseline population data were collected the DOTIFS scale could then be used as a reactionary and quickly deployed assessment of consumer trust following a food safety incident or scandal, and the different domains would then assist in developing sociological theory regarding what areas of trust are impacted by such events, and therefore where to invest resources to best support trust in these situations. Similarly, the DOTIFS scale could be used to assess the impact of new policy and regulatory decisions relating to food, such as the introduction of the Health Star Rating food labelling system, or in consumer research examining different policy scenarios during decision making.

More widely, the DOTIFS scale has a number of features not found in similar scales. For example, Benson et al. [33] conceptualise trust wholly differently to the sociological conceptualisation found here, citing trust as a personality trait, and defining trust and distrust as separate factors within their scale, which has an almost exclusive focus on the food chain and food safety. The DOTIFS scale however aims to capture the broad and interrelated aspects of the whole food system as seen and interpreted by consumers, and as is therefore consistent with a sociological conceptualisation of trust. The survey by Poppe and Kjaernes [31] which explored trust in food in Europe was not self-completed and could not easily be deployed quickly to calibrate food trust at strategic times, e.g., during a food scare. The DOTIFS scale has both capabilities. However mindful that the window on public trust is often short-lived and limited, and the DOTIFS scale requires a considerable time investment from participants, further research could also explore the potential to extract a short-form version of the DOTIFS scale from that presented here. This could be incorporated regularly into the recurring large scale global assessments of general, not food specific, trust already being conducted [13, 14, 25, 27, 30]. This would represent a considerable step forward in the monitoring of consumer trust globally, and potentially lead to international comparisons which, when explored using methodological approaches such as comparative health research [54], could result in significant new insights into how consumer trust in food systems is built, broken and repaired.

## 5. Conclusion

Finally, returning to the aims and objectives of this research we believe that we have been able to develop a comprehensive, sociologically informed, self-completed instrument to measure consumer trust in the food system: the DOTIFS scale. Further, we have been able to show that the DOTIFS scale can be demonstrates differences in adherence to national dietary guidelines, exposure to public health fortification programs, and adherence to recommended food safety practices associated with trust in the food system.

## Supplementary Information

**Additional file 1.**

## Data Availability

The datasets generated and analysed during the current study are not publicly available due ethical reasons but are available from the corresponding author on reasonable request.

## Abbreviations

CSIRO: Commonwealth Scientific and Industrial Research Organisation
DOTIFS: The Dimensions of Trust in Food Systems Scale
FSANZ: Food Standards Australia New Zealand
OECD: Organisation for Economic Co-operation and Development
