# Supplementary material for: The health implications of distrust in the food system: findings from the dimensions of trust in food systems scale (DOTIFS scale)
Source: BMC Public Health. 2021 Jul 28;21:1468. doi: 10.1186/s12889-021-11349-9 (PMC8317288; doi:10.1186/s12889-021-11349-9)
Supplement: Supplementary file 1 — Additional file 1. [file 12889_2021_11349_MOESM1_ESM.docx]

# Appendix A: The Dimensions of Trust in Food Systems Scale (DOTIFS scale)

**Domain 1. Generalised Trust**

These questions ask about your trust in people in general, not specifically about food.

The first questions are about your trust in other people as individuals.

Q1. In general, how much do you trust most people?

| Not at all |  | | | | | Completely |
| --- | --- | --- | --- | --- | --- | --- |
| 1 | 2 | 3 | 4 | 5 | 6 | 7 |

Q2. In general, how much do you trust most people you know personally?

| Not at all |  | | | | | Completely |
| --- | --- | --- | --- | --- | --- | --- |
| 1 | 2 | 3 | 4 | 5 | 6 | 7 |

The next questions are about whether you have trust in various institutions and organisations in Australia. Even if you have had very little or no contact with these institutions, please base your answer on your general impression of them.

Q3. How much do you personally trust the following organisations or institutions?

|  | Not at all |  | | | | | Completely |
| --- | --- | --- | --- | --- | --- | --- | --- |
| Religious organisations | 1 | 2 | 3 | 4 | 5 | 6 | 7 |
| The legal system | 1 | 2 | 3 | 4 | 5 | 6 | 7 |
| The media | 1 | 2 | 3 | 4 | 5 | 6 | 7 |
| The Federal Government | 1 | 2 | 3 | 4 | 5 | 6 | 7 |
| The police | 1 | 2 | 3 | 4 | 5 | 6 | 7 |
| Banks | 1 | 2 | 3 | 4 | 5 | 6 | 7 |

**Domain 2: Food Safety and Integrity**

In talking about the quality of food, people often refer to both its safety in terms of health, as well as whether it was made in a way that aligns with their consumer values. Consumer values can be thought of as the beliefs you hold about the way food should be produced (for example, considering fair trade, animal welfare or environmental sustainability). The following questions ask separately about food safety and consumer values.

Q4. How confident are you that all food sold in Australian shops and supermarkets is safe?

| Not at all confident |  | | | | | Completely confident |
| --- | --- | --- | --- | --- | --- | --- |
| 1 | 2 | 3 | 4 | 5 | 6 | 7 |

Q5. How confident are you that the foods you buy for your household are safe?

| Not at all confident |  | | | | | Completely confident |
| --- | --- | --- | --- | --- | --- | --- |
| 1 | 2 | 3 | 4 | 5 | 6 | 7 |

Q6. How much do you believe that the following foods are safe?

|  | Not at all safe |  | | | | | Completely safe |
| --- | --- | --- | --- | --- | --- | --- | --- |
| Fresh foods grown in Australia | 1 | 2 | 3 | 4 | 5 | 6 | 7 |
| Fresh foods from a farmers’ market | 1 | 2 | 3 | 4 | 5 | 6 | 7 |
| Foods imported to Australia | 1 | 2 | 3 | 4 | 5 | 6 | 7 |
| Packaged foods at a farmers’ market | 1 | 2 | 3 | 4 | 5 | 6 | 7 |
| Organic foods | 1 | 2 | 3 | 4 | 5 | 6 | 7 |
| Foods at a community garden | 1 | 2 | 3 | 4 | 5 | 6 | 7 |
| Fresh foods from a grocer | 1 | 2 | 3 | 4 | 5 | 6 | 7 |
| Packaged foods at a supermarket | 1 | 2 | 3 | 4 | 5 | 6 | 7 |
| Fresh foods from a supermarket | 1 | 2 | 3 | 4 | 5 | 6 | 7 |

Q7. How much do you think that all food sold in Australian shops and supermarkets reflects your consumer values?

| Not at all |  | | | | | Completely |
| --- | --- | --- | --- | --- | --- | --- |
| 1 | 2 | 3 | 4 | 5 | 6 | 7 |

Q8. How much do you think that the foods you buy for your household reflect your consumer values?

| Not at all |  | | | | | Completely |
| --- | --- | --- | --- | --- | --- | --- |
| 1 | 2 | 3 | 4 | 5 | 6 | 7 |

Q9. In general, when buying or eating food, how often do you feel confident that it is what it says it is on the label?

| Not at all |  | | | | | Completely |
| --- | --- | --- | --- | --- | --- | --- |
| 1 | 2 | 3 | 4 | 5 | 6 | 7 |

*ValidityQuestion1. The last section aimed to measure your trust in the safety and integrity of food. Your score for this section is XX on a scale from 1 = complete distrust to 7 = complete trust, and 4 is the middle.*

*How accurately does your score capture this for you?*

| *Not at all accurate* | *Not very accurate* | *Reasonably accurate* | *Completely accurate* |
| --- | --- | --- | --- |
| *□* | *□* | *□* | *□* |

**Domain 3: Trust in Food System Organisations and Institutions**

The following questions ask about your trust in the people and organisations that make up the Australian food system.

Q10. How much do you **trust** the following food system people and institutions to **do what is right**?

|  | Do not trust them at all |  | | | | | Trust them completely |
| --- | --- | --- | --- | --- | --- | --- | --- |
| Press, television and radio | 1 | 2 | 3 | 4 | 5 | 6 | 7 |
| Supermarket chains | 1 | 2 | 3 | 4 | 5 | 6 | 7 |
| Small food companies | 1 | 2 | 3 | 4 | 5 | 6 | 7 |
| Farmers | 1 | 2 | 3 | 4 | 5 | 6 | 7 |
| Stall holders at farmers’ markets | 1 | 2 | 3 | 4 | 5 | 6 | 7 |
| Consumer organisations | 1 | 2 | 3 | 4 | 5 | 6 | 7 |
| Politicians | 1 | 2 | 3 | 4 | 5 | 6 | 7 |
| Government/public food authorities | 1 | 2 | 3 | 4 | 5 | 6 | 7 |
| Food producers you know personally | 1 | 2 | 3 | 4 | 5 | 6 | 7 |
| Large food companies | 1 | 2 | 3 | 4 | 5 | 6 | 7 |
| Health professionals | 1 | 2 | 3 | 4 | 5 | 6 | 7 |
| Scientists | 1 | 2 | 3 | 4 | 5 | 6 | 7 |

Here when we say 'food companies' we are referring to the industry that makes and sells fresh and packaged food in Australia, from producers through to supermarkets and retailers. We are not talking about the hospitality sector.

Q11. How much do you agree with the following statements about **food companies**?

|  | Do not agree at all |  | | | | | Completely agree |
| --- | --- | --- | --- | --- | --- | --- | --- |
| I trust food companies to do what is right | 1 | 2 | 3 | 4 | 5 | 6 | 7 |
| Food companies are good at what they do | 1 | 2 | 3 | 4 | 5 | 6 | 7 |
| Food companies are trying hard to have a positive impact on society | 1 | 2 | 3 | 4 | 5 | 6 | 7 |
| Food companies keep their promises | 1 | 2 | 3 | 4 | 5 | 6 | 7 |
| Food companies are honest | 1 | 2 | 3 | 4 | 5 | 6 | 7 |

The following questions are about your expectations of behaviour from the public institutions who govern and regulate Australia's food supply.

Q12. If you were to make a complaint about how food in Australia is made or sold, how likely is it that the problem would be easily resolved?

| Very likely |  | | | | | Very unlikely |
| --- | --- | --- | --- | --- | --- | --- |
| 1 | 2 | 3 | 4 | 5 | 6 | 7 |

Q13. In the event of a food incident, how likely do you think it is that government would manage it in a timely and efficient way?

| Very likely |  | | | | | Very unlikely |
| --- | --- | --- | --- | --- | --- | --- |
| 1 | 2 | 3 | 4 | 5 | 6 | 7 |

Q14. If a decision affecting the way Australian food is made or sold were to be taken by the government, how likely is it that you and others in the community would have an opportunity to voice your concerns?

| Very likely |  | | | | | Very unlikely |
| --- | --- | --- | --- | --- | --- | --- |
| 1 | 2 | 3 | 4 | 5 | 6 | 7 |

Q15. If the cost of food regulation increased, how likely is it that government would ensure the financial burden was shared fairly between consumers and food companies?

| Very likely |  | | | | | Very unlikely |
| --- | --- | --- | --- | --- | --- | --- |
| 1 | 2 | 3 | 4 | 5 | 6 | 7 |

Q16. If a large food company lobbied the government about a food regulation decision, how likely do you think it would be that this lobbying influenced the government's decision?

| Very likely |  | | | | | Very unlikely |
| --- | --- | --- | --- | --- | --- | --- |
| 1 | 2 | 3 | 4 | 5 | 6 | 7 |

*ValidityQuestion2. The last section aimed to measure your trust in the organisations that are responsible for food in Australia. Your score for this section is XX on a scale from 1 = complete distrust to 7 = complete trust, and 4 is the middle.*

*How accurately does your score capture this for you?*

| *Not at all accurate* | *Not very accurate* | *Reasonably accurate* | *Completely accurate* |
| --- | --- | --- | --- |
| *□* | *□* | *□* | *□* |

**Domain 4: Food Concerns**

Some people say they worry about many things, while others say they have few concerns. We are interested in how much you worry about food related concerns.

Q17. To what extent do you believe it is important for you to personally monitor the safety and quality of food?

| Not at all important |  | | | | | Extremely important |
| --- | --- | --- | --- | --- | --- | --- |
| 1 | 2 | 3 | 4 | 5 | 6 | 7 |

Q18. How much do you worry about the following?

|  | Not at all worried |  | | | | | Extremely worried |
| --- | --- | --- | --- | --- | --- | --- | --- |
| Food waste | 1 | 2 | 3 | 4 | 5 | 6 | 7 |
| Food poisoning, like salmonella | 1 | 2 | 3 | 4 | 5 | 6 | 7 |
| Pollution/environmental issues | 1 | 2 | 3 | 4 | 5 | 6 | 7 |
| Environmental sustainability in food production | 1 | 2 | 3 | 4 | 5 | 6 | 7 |
| Added sugar in food | 1 | 2 | 3 | 4 | 5 | 6 | 7 |
| General crime levels | 1 | 2 | 3 | 4 | 5 | 6 | 7 |
| Food not being what the label says it is | 1 | 2 | 3 | 4 | 5 | 6 | 7 |
| Allergens in food | 1 | 2 | 3 | 4 | 5 | 6 | 7 |
| Chemicals from the environment in food, like toxic metals from pollution | 1 | 2 | 3 | 4 | 5 | 6 | 7 |
| Food miles, the distance food travels | 1 | 2 | 3 | 4 | 5 | 6 | 7 |
| The use of additives, like preservatives | 1 | 2 | 3 | 4 | 5 | 6 | 7 |
| Hormones/steroids/antibiotics in food | 1 | 2 | 3 | 4 | 5 | 6 | 7 |
| The use of pesticides to grow food | 1 | 2 | 3 | 4 | 5 | 6 | 7 |
| Artificial sweeteners | 1 | 2 | 3 | 4 | 5 | 6 | 7 |
| Unreasonable food prices | 1 | 2 | 3 | 4 | 5 | 6 | 7 |
| Household finances | 1 | 2 | 3 | 4 | 5 | 6 | 7 |
| Animal welfare | 1 | 2 | 3 | 4 | 5 | 6 | 7 |
| Cancer-causing chemicals in food | 1 | 2 | 3 | 4 | 5 | 6 | 7 |
| Terrorism | 1 | 2 | 3 | 4 | 5 | 6 | 7 |
| Genetically modified foods | 1 | 2 | 3 | 4 | 5 | 6 | 7 |
| Fair trade in food production | 1 | 2 | 3 | 4 | 5 | 6 | 7 |

*ValidityQuestion3. The last section aimed to measure how much you are concerned about the safety and quality of Australian food in a broad sense. Your score is XX on a scale from 1 = extremely worried to 7 = not at all worried, and 4 is the middle.*

*How accurately does your score capture this for you?*

| *Not at all accurate* | *Not very accurate* | *Reasonably accurate* | *Completely accurate* |
| --- | --- | --- | --- |
| *□* | *□* | *□* | *□* |

**Domain 5: Engagement with Food Issues and Activism**

We would now like to get a picture of how much time and energy you put into action on concerns about food you may have.

Q19. Have you ever done any of the following because of concerns you have about food?

|  | Yes | No |
| --- | --- | --- |
| Read food labels more often? | □ | □ |
| Stopped/started shopping for food at certain place? | □ | □ |
| Tried to get more information about a food concern? | □ | □ |
| Changed the way you cook food? | □ | □ |
| Changed the way you prepare food (including washing)? | □ | □ |
| Looked up safe ways to handle food? | □ | □ |

Q20. Have you been involved in any of the following situations over the past year?

|  | Yes | No |
| --- | --- | --- |
| Complained to a retailer about food quality? | □ | □ |
| Refused to buy certain foods or brands to express your opinion on a political or social issue? | □ | □ |
| Bought particular foods or brands in order to encourage or support a political or social issue? | □ | □ |
| Participated in organised consumer boycotts? | □ | □ |
| Been a member of an organisation that works for the improvement of food? | □ | □ |
| Taken part in any kind of public action in order to improve the food supply (contacted a politician, signed a petition, supported a campaign with money, distributed leaflets, participated in demonstration)? | □ | □ |

*ValidityQuestion4. The last section aimed to get an idea of the energy you put into action on food concerns you may have and how much you participate in activism around this. Your score is XX on a scale from 1 = extremely engaged to 7 = not at all engaged, and 4 is the middle.*

*How accurately does your score capture this for you?*

| *Not at all accurate* | *Not very accurate* | *Reasonably accurate* | *Completely accurate* |
| --- | --- | --- | --- |
| *□* | *□* | *□* | *□* |

**Domain 6: Belief in the Food System**

Q21. Below is a list of statements which we would like you to apply to the food system in Australia. How true do you believe each statement to be?

|  | Not true at all |  | | | | | Completely true |
| --- | --- | --- | --- | --- | --- | --- | --- |
| Australia’s food system will be better for Australia’s children than it is for me | 1 | 2 | 3 | 4 | 5 | 6 | 7 |
| Those who run Australia’s food system are out of touch with regular people | 1 | 2 | 3 | 4 | 5 | 6 | 7 |
| Those who run Australia’s food system are indifferent to the will of the Australian people | 1 | 2 | 3 | 4 | 5 | 6 | 7 |
| As regular people work to make ends meet, those who run Australia’s food system are getting richer than they deserve | 1 | 2 | 3 | 4 | 5 | 6 | 7 |
| Australia’s food system is moving in the right direction | 1 | 2 | 3 | 4 | 5 | 6 | 7 |
| Australia’s food system is biased against regular people and in favour of the rich and powerful | 1 | 2 | 3 | 4 | 5 | 6 | 7 |
| I have confidence that Australia’s current food system leader will be able to address Australia’s food system challenges | 1 | 2 | 3 | 4 | 5 | 6 | 7 |
| We need forceful reformers in positions of power to bring about much needed change in Australia’s food system | 1 | 2 | 3 | 4 | 5 | 6 | 7 |

*ValidityQuestion5. The last section aimed to measure your fundamental belief in the food system in Australia as an institution. Your score is XX on a scale from 1 = do not have any confidence in it to 7 = fully confident in it, and 4 is the middle.*

*How accurately does your score capture this for you?*

| *Not at all accurate* | *Not very accurate* | *Reasonably accurate* | *Completely accurate* |
| --- | --- | --- | --- |
| *□* | *□* | *□* | *□* |

*ValidityQuestion6. If you had to score your level of trust in the Australian food system after completing this survey and considering all the different types of things we asked about in it, would it be:*

- Strongly trust
- Mostly trust
- Undecided, on the fence
- Mostly do not trust
- Strongly do not trust

***-- section break, respondents could not go back and change answers from this point --***

*ValidityQuestion7. Your overall trust in the food system score is XX/30, where 0 is complete distrust, 15 is the middle, and 30 is complete trust.*

*How accurately does this overall score reflect your trust in the food system?*

| *Not at all accurate* | *Not very accurate* | *Reasonably accurate* | *Completely accurate* |
| --- | --- | --- | --- |
| *□* | *□* | *□* | *□* |

**Domain 7: Sociodemographic questions**

Where do you predominantly shop for food?

- Grow my own
- Supermarket (eg Coles, Woolworths, Aldi, Costco)
- Farmers’ market
- Small specialty stores (eg grocer, butcher, bakery, organic stores)
- Community gardens
- Online box deliveries (eg Hello Fresh, Lite and Easy)

What is your age?

- 18-24
- 25-34
- 35-44
- 45-54
- 55-64
- 65+

To what gender do you most identify?

- Female
- Male
- Prefer not to say

What is your current marital status?

- Single (never married)
- Married/defacto
- Widowed
- Divorced
- Separated

What is the highest level of formal education you have completed?

- Year 11 or below
- Secondary school
- Diploma/vocational training
- Bachelor’s degree
- Higher degree

Which of the following best describes where you live?

- Family household
- Singe person household
- Group/share house
- Other: _________________________

Are there children under 12 year in your household?

- Yes
- No

Which of the following best describes you?

- Born in Australia
- Born outside of Australia in a primarily English language country
- Born outside of Australia in a primarily non-English language country

Do you usually think of yourself as close to any particular political party, and if yes, which party is that?

- Labour (ALP)
- Liberal Party
- National (Country) Party
- Greens
- None of these
- No party affiliation

Please select all the types of media coverage you engage with

- Television – commercial (Ten, Seven, Nine, Foxtel)
- Television – independent (ABC)
- Streaming (Netflix, Stan)
- Radio – commercial (Triple M, Mix, Nova)
- Radio – independent (Triple J, Radio National)
- Facebook
- Twitter
- Instagram
- Print media – magazines
- Print/online news media – tabloid (The Advertiser)
- Print/online news media- broadsheet (The Australian)
- Independent online news (ABC news)

Do any of the following apply to you or any members of your household?

- Watching my/others’ weight generally
- Digestive concerns such as coeliac disease or IBS
- Food allergy
- Diabetes
- Heart disease
- On a specific diet
- Vegetarian/vegan
- Religious/ethical beliefs that influence dietary choices
- None of these

Which of the following best describe where you live?

- Major city (Adelaide and surrounding suburbs)
- Inner regional (Adelaide Hills, Fleurieu Peninsula)
- Outer regional (Port Augusta, Mount Gambier)
- Remote (Port Lincoln)
- Very remote (Northern SA)

We are so grateful for your time.

Thank you for helping us to further the understanding of trust in Australia's food system.
